# Association between inflammatory cytokines in the aqueous humor and hyperreflective foci on optical coherence tomography in patients with neovascular age-related macular degeneration and polypoidal choroidal vasculopathy

**DOI:** 10.3389/fmed.2022.973025

**Published:** 2022-09-23

**Authors:** Jianbo Mao, Nuo Chen, Shian Zhang, Yuyan Fang, Zicheng Zheng, Sulan Wu, Xin Ye, Yijing Chen, Yiqi Chen, Lijun Shen

**Affiliations:** ^1^Center for Rehabilitation Medicine, Department of Ophthalmology, Zhejiang Provincial People's Hospital, Hangzhou, China; ^2^School of Ophthalmology and Optometry, Wenzhou Medical University, Wenzhou, China; ^3^Hwa Mei Hospital, University of Chinese Academy of Sciences, Ningbo, China

**Keywords:** aqueous humor, cytokine, hyperreflective foci, neovascular age-related macular degeneration, polypoidal choroidal vasculopathy, spectral-domain optical coherence tomography

## Abstract

**Purpose:**

To investigate the associations between cytokine levels in the aqueous humor (AH) and hyperreflective foci (HF) on spectral-domain optical coherence tomography (SD-OCT) in neovascular age-related macular degeneration (nAMD) and polypoidal choroidal vasculopathy (PCV).

**Methods:**

The prospective study included 63 eyes with nAMD, 44 with PCV, and 43 with cataracts (Controls). AH samples were obtained before anti-vascular endothelial growth factor (VEGF) therapy or cataract surgery. Cytokines interleukin 6 (IL-6), IL-8, IL-10, interferon-inducible protein 10 (IP-10), monocyte chemotactic protein 1 (MCP-1), and VEGF were measured by multiplex bead assay. Best-corrected visual acuity (BCVA), central macular thickness (CMT), and the number of HF were evaluated at baseline and 1 month after anti-VEGF treatment.

**Results:**

No significances difference in IL-6 and IL-8 levels were noted among the three groups (*P* = 0.370 and *P* = 0.067). VEGF, IP-10, and IL-10 levels were significantly higher in nAMD and PCV groups than in Controls (all *P* < 0.05). In nAMD, HF was positively correlated with VEGF (*r*_*s*_ = 0.300, *P* = 0.025) and in eyes with HF group, VEGF and IL-10 were significantly higher than those without HF (*P* = 0.008 and *P* = 0.022). In PCV, no correlation was observed between HF and cytokines (all *P* > 0.05). After anti-VEGF treatment, patients with HF in nAMD and PCV were predisposed to worse visual outcomes (*P* = 0.022 and *P* = 0.015) and a significantly greater reduction in CMT (*P* = 0.001 and *P* = 0.057). And nAMD patients with HF were more sensitive to anti-VEGF treatment than those without HF (*P* = 0.029).

**Conclusions:**

In the nAMD group, HF was positively correlated with VEGF. Patients in nAMD with HF had elevated levels of VEGF and IL-10 and responded favorably to anti-VEGF. HF might serve as an inflammatory biomarker and a predictive factor for therapeutic efficacy in patients with nAMD.

## Introduction

Age-related macular degeneration (AMD) is a progressive chronic disease that causes visual impairment and severe vision loss (1). It is estimated that the global prevalence of AMD is 190 million persons by 2020, and will increase to 288 million persons in 2040 (2). AMD is mainly divided into dry (also known as non-vascular, non-exudative and atrophic) and wet (also known as neovascular and exudative) forms, with wet AMD causing the most severe vision loss (1, 2). Anti-VEGF therapy is now the most commonly used treatment for AMD with satisfactory outcomes. Polypoidal choroidal vasculopathy (PCV), a vascular disease of the choroid, is considered to be a common subtype of nAMD. However, recent studies in the fields of genetics, proteomics, and imaging have further clarified the distinction between nAMD and PCV (3). Previous research demonstrated that inflammation was likely to play a role in the pathogenesis of AMD and PCV (4, 5). Various local and systemic inflammatory molecules, including cytokines, have been proposed as biomarkers of AMD but at present, no specific and reliable marker has been found (6).

Hyperreflective foci (HF) was visualized as discrete, well-circumscribed lesions with greater reflectivity than the retinal pigment epithelium (RPE) band on spectral-domain optical coherence tomography (SD-OCT), which was considered a structural biomarker associated with disease progression, treatment response, and prognosis of several retinal diseases, including AMD (7), diabetic macular edema (8) and central serous chorioretinopathy (9). The origin of HF is not clear, but many studies supported the hypothesis that HF was as aggregates of activated microglial cells in the retina and possible *in vivo* biomarker of local inflammation (10, 11). Preliminary studies had shown that HF was correlated with complement factors upregulation in aqueous humor (AH) of patients with early stages of AMD (12). And the presence of HF might reflect a degree of local inflammation including complement activation in the eye (12). However, the association between HF and intraocular cytokines of patients with AMD and PCV was rarely reported. Better understanding of the relationship between HF and cytokines in AH of patients with AMD and PCV could provide new insights into the distinct pathophysiological processes between AMD and PCV. And this may provide individualized therapeutic strategies and prognosis of diseases.

In this study, we investigated the associations between AH concentrations of inflammatory cytokines and the number of HF on SD-OCT in patients with nAMD and PCV. Moreover, we evaluated the treatment responses to anti-VEGF injections in nAMD and PCV eyes with or without HF to explore the role of HF in treatment efficacy.

## Materials and methods

This prospective study was reviewed and performed at Eye Hospital of Wenzhou Medical University between May 2019 and January 2021, and the study protocol (No. 121-K107-01) was approved by the ethics committee of The Eye Hospital of Wenzhou Medical University. All of the procedures adhered to the tenets of the Declaration of Helsinki. Written informed consent was obtained from all subjects.

### Patients

The subjects included 63 with nAMD (44 males, 19 females), 44 patients with PCV (28 males, 16 females), and 43 controls (14 males, 29 females) without any sight-threatening disease except for cataract who were scheduled for cataract surgery.

The inclusion criteria were (1) Patients with nAMD and PCV were diagnosed specifically with fluorescein angiography (FA) and indocyanine green angiography (ICGA), (2) nAMD and PCV patients were treatment-naive and scheduled to receive the anti-VEGF intravitreal injection, (3) central macular thickness (CMT) >250 μm, and (4) Patients aged over 18 years. The exclusion criteria were (1) Subjects who have undergone intraocular surgery or procedures within the last 6 months, (2) Patients who were diagnosed with systemic inflammatory medical conditions or malignancies, and (3) Patients suffering from other eye disorders such as diabetic retinopathy, retinal vascular occlusion or retinal detachment except for the included diseases in the study. The differential diagnosis of nAMD and PCV was based mainly on the ICGA findings. Eyes with nAMD showed classic choroidal neovascularization (CNV) or occult CNV with FFA, with no polypoidal lesions in ICGA. According to the origin and location of neovascular vessels, CNV is classified as type 1 CNV (beneath the RPE) and type 2 CNV (above the RPE) in the nAMD group (13). PCV presents as polypoid vasodilation with or without superficial choroidal vascular abnormalities.

Before anti-VEGF injection or cataract surgery, all patients underwent an extensive ophthalmologic examination that included best-corrected distance visual acuity (recorded as the logarithm of the minimum angle of resolution, logMAR), slit-lamp biomicroscopy, intraocular pressure measurement, and dilated funduscopic examination.

### Anti-VEGF therapy and aqueous humor sample collection

Patients initially diagnosed with nAMD or PCV who were in the acute phase received the anti-VEGF drugs ranibizumab (0.5 mg/0.05 ml, Lucentis; Novartis Pharma AG, Basel, Switzerland) or conbercept (0.5 mg/0.05 ml, KH902; Biotech Co., Ltd., Sichuan, China) therapy. Retreatment or change of treatment regimen was considered according to clinical response at a 1-month follow-up. AH samples were collected before cataract surgery in the control group or before intravitreal anti-VEGF drugs (ranibizumab or conbercept) injection into the eyes with nAMD or PCV. After topical anesthesia, ~0.05–0.1 ml of AH was withdrawn at the corneal limbus using a tuberculin syringe with a 30-gauge needle. The samples were immediately frozen and stored at −80°C until analysis of the cytokines. Luminex200 (BIO-RAD, Hercules, CA, USA) was used for the detection of VEGF, interleukin 6 (IL-6), IL-8, IL-10, interferon-inducible protein 10 (IP-10), and monocyte chemotactic protein 1 (MCP-1). The concentration of AH cytokine was calculated and presented as pg/ml from the standard curve of each specific cytokine provided by the kit (LXSAHM-06, RnD).

### Examination protocol and outcome measures of SD-OCT

All patients with nAMD and PCV were imaged using Heidelberg Spectralis (Heidelberg Engineering, Heidelberg, Germany) before intravitreal anti-VEGF injections in the nAMD and PCV groups. A volume scan comprising 18 horizontal B-scans covering a 6 × 6 mm area of the macula region within the fovea centered was obtained using SD-OCT. Central macular thickness (CMT) was automatically calculated as the average retinal thickness within a circle having a 500-mm radius, centered on the fovea, based on the volume scan data containing the target circle area. The change in CMT (ΔCMT) at 1 month following anti-VEGF injection was calculated by subtracting the post-injection thickness from the baseline thickness. The CMT_reduction_ratio_ was calculated as the ratio of ΔCMT to the remaining CMT, which itself was calculated as the baseline CMT minus 250 μm: (14, 15).


$$\begin{array}{l} CMT_{\text{reduction\_ratio}}=\frac{\left(CMTbaseline\;-\;CMTpost-treatment\right)}{CMTbaseline-250\mu m} \end{array}$$

The CMT_reduction_ratio_ responses to anti-VEGF therapy were classified as “sensitive” in which the CMT_reduction_ratio_ was ≥30% or as “non-sensitive” in which the CMT_reduction_ratio_ was < 30%.

**Figure 1:**
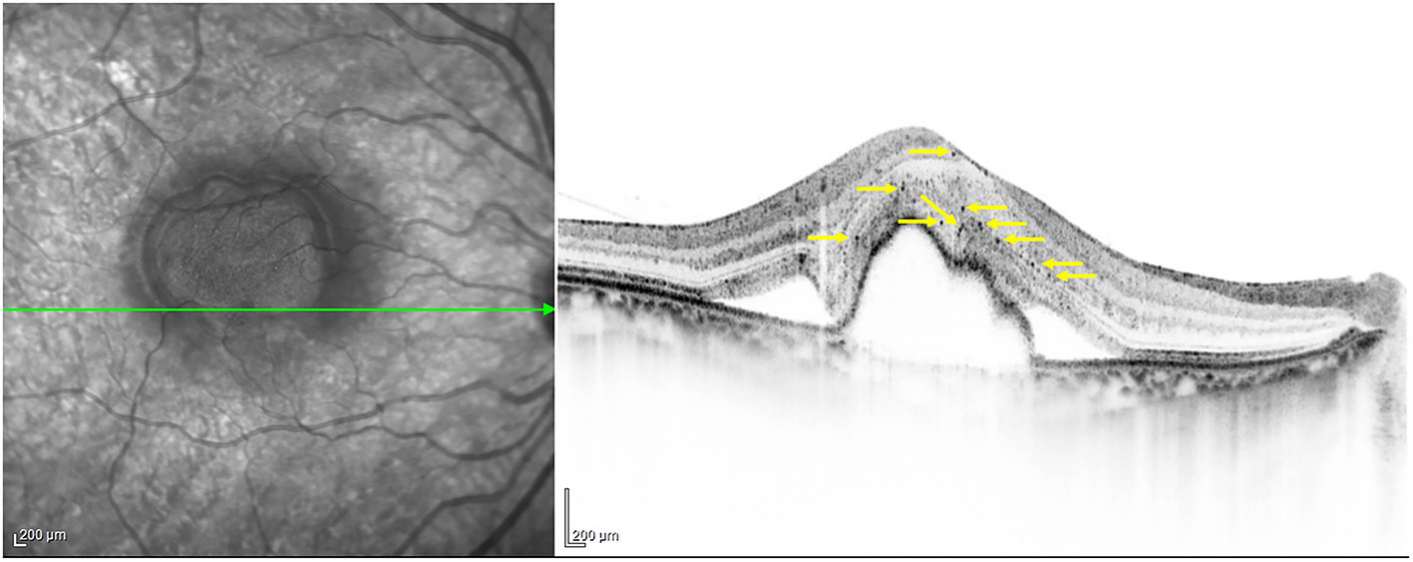
HF, hyperreflective foci indicated by yellow arrows.

nAMD or PCV eyes were divided into two groups based on the presence of HF, that is, HF positive (+) group and HF negative (–) group for analysis of the clinical characteristics. The presence of HF was defined as the presence of small focal hyperreflective material (as hyperreflective as RPE) observed in at least one available scan (16). The number of HF was manually counted within the 6-mm area centered on the fovea in the fovea-spanning horizontal raster scan (Figure 1). Two independent observers separately counted the number of HF, and the values averaged. The diameter of HF was limited to within a range of 20–50 μm to exclude counting small noise signals as HF and to prevent the inclusion of large confluent HF clumps, which are present as typical hard exudates in fundus photography.

### Statistical analysis

All statistical analyses were performed using SPSS 26.0 for Windows (SPSS Inc., Chicago, IL, USA). Quantitative variables were tested for normal distribution by the Shapiro–Wilk test. Variables displaying skewed distribution were expressed as the median and interquartile range (IQR). Groups of discrete variables were compared by means of the Mann–Whitney *U* test or Kruskal–Wallis nonparametric analysis of variance. For the Kruskal–Wallis test, *P*-values were adjusted by the Bonferroni correction for multiple comparisons of continuous variables. Categorical variables were analyzed using the Chi-square test. Spearman rho tests were used for correlation analyses. A probability value (*P*-value) < 0.05 was considered statistically significant.

## Results

The average age of the patients with nAMD, PCV, and control was 70.0 (IQR, 61.0–82.0), 67.0 (IQR, 61.3–75.0), and 72.0 years (IQR, 64.0–76.0), respectively (Table 1). There was no statistical difference in mean age among the three groups (*P* = 0.200, Table 1). The male and female ratios were 44:19 and 28:16 in nAMD and PCV patients, respectively, and 14:29 in controls. Gender showed statistical difference among the three groups (*P* < 0.001). However, no significant differences were noted in six cytokines levels between male and female (all *P* > 0.05, Supplementary Table S1).

**Table 1:** Baseline characteristics among three groups.

**Characteristics**	**nAMD group**	**PCV group**	**Control group**	***X*^2^/*Z***	***P***
Age, years	70.0 (61.0–82.0)	67.0 (61.3–75.0)	72.0 (64.0–76.0)	3.22	0.200^a^
Sex, M/F	44/19	28/16	14/29	15.53	< 0.001^b^
IL-6, pg/ml	3.09 (1.74–5.58)	2.98 (1.98–4.94)	4.79 (1.63–9.54)	1.99	0.370^a^
VEGF, pg/ml	39.30 (24.61–63.61)	37.93 (26.46–48.11)	27.91 (20.72–36.26)	12.38	0.002^a^
IP-10, pg/ml	339.12 (203.49–493.34)	528.37 (340.51–914.61)	148.10 (112.03–238.79)	53.35	< 0.001^a^
MCP-1, pg/ml	415.59 (323.09–488.61)	492.44 (392.09–649.53)	404.22 (332.11–547.42)	7.91	0.019^a^
IL-8, pg/ml	8.79 (6.14–15.77)	10.10 (7.48–23.39)	8.65 (4.95–13.16)	5.39	0.067^a^
IL-10, pg/ml	0.94 (0.73–1.16)	0.88 (0.79–1.45)	0.63 (0.53–0.88)	26.28	< 0.001^a^
BCVA (logMAR)	0.70 (0.40–1.00)	0.70 (0.40–1.08)	0.40 (0.30–0.80)	5.54	0.063^a^
CMT (μm)	322.00 (262.00–456.00)	415.50 (323.00–539.00)	/	889.00	0.002^c^
HF (*n*)	1.5 (0–3)	2.0 (0–4)	/	1229.00	0.677^c^

Data are the median [interquartile range, (IQR)] and frequency. ^*a*^Kruskal–Wallis test. ^*b*^Chi-square test. ^*c*^Mann–Whitney U-test. IL-6, interleukin 6; VEGF, vascular endothelial growth factor; IP-10, interferon-inducible protein 10; MCP-1, monocyte chemotactic protein 1; IL-8, interleukin 8; IL-10, interleukin 10; BCVA, best-corrected visual acuity; logMAR, log of the minimal angle of resolution; CMT, central macular thickness; HF, hyper-reflective foci.

### Baseline differences in cytokine levels and SD-OCT structural parameters among the three groups

There were no significant differences in the levels of IL-6 and IL-8 among the three groups (*P* = 0.370 and *P* = 0.067, Table 1). The levels of VEGF (*P* = 0.002 and *P* = 0.025), IP-10 (*P* < 0.001 and *P* < 0.001), and IL-10 (*P* < 0.001 and *P* < 0.001) in nAMD and PCV groups were significantly higher than in controls. And MCP-1 levels were significantly elevated in the PCV group than in nAMD group (*P* = 0.021), but they did not differ from the control group (*P* = 0.102).

Based on baseline measurements of BCVA and SD-OCT parameters, there was no significant difference in BCVA among the three groups (*P* = 0.063, Table 1). CMT baseline value was significantly higher in the PCV group than in nAMD group (*P* = 0.002); however, the number of HF in patients with nAMD and PCV was not different (*P* = 0.677).

### Baseline differences between nAMD and PCV groups in cytokine concentrations in eyes with or without HF

The AH levels of VEGF and IL-10 [median: 47.93 and 1.05 pg/ml, respectively, interquartile range (IQR), 32.13–64.47 and 0.82–1.22, respectively] were significantly higher in the nAMD patients with HF compared to those without HF (*P* = 0.008 and *P* = 0.022, respectively, Table 2). There were no significant differences in concentrations of AH cytokines IL-6, IP-10, MCP-1, or IL-8 between the nAMD patients with HF and without HF (all *P* > 0.05). Similarly, none of the cytokine levels investigated in the present study were different between the PCV patients with HF and without HF groups (all *P* > 0.05, Table 2).

**Table 2:** AH concentrations (pg/mL) of cytokines, BCVA, and SD-OCT parameters in eyes with or without HF in the nAMD and PCV groups.

	**nAMD group**		***Z***	***P***	**PCV group**		***Z***	***P***
	**HF (–) (*n* = 22)**	**HF (+) (*n* = 41)**			**HF (–) (*n* = 16)**	**HF (+) (*n* = 28)**		
	**Median (IQR)**	**Median (IQR)**			**Median (IQR)**	**Median (IQR)**		
IL-6, pg/mL	3.08 (2.44–4.31)	3.20 (1.73–6.11)	427.50	0.983	3.35 (1.59–5.55)	2.52 (2.03–3.58)	192.50	0.442
VEGF, pg/mL	27.82 (18.33–44.97)	47.93 (32.13–64.47)	232.50	0.008	38.50 (27.94–50.06)	37.68 (21.61–46.85)	167.50	0.554
IP-10, pg/mL	262.54 (152.28–469.57)	380.62 (228.11–514.80)	351.50	0.318	538.89 (349.29–967.55)	509.39 (314.43–754.19)	199.00	0.542
MCP-1, pg/mL	415.57 (297.09–473.94)	429.34 (358.81–489.69)	395.50	0.688	499.14 (409.45–678.60)	468.25 (346.65–567.47)	178.00	0.262
IL-8, pg/mL	6.93 (5.15–14.30)	9.40 (6.75–15.87)	326.50	0.111	10.30 (7.49–23.04)	8.41 (7.27–21.99)	199.00	0.669
IL-10, pg/mL	0.79 (0.62–1.05)	1.05 (0.82–1.22)	133.00	0.022	0.88 (0.78–1.58)	0.97 (0.79–1.42)	115.50	0.864
BCVA_baseline_	0.61 (0.30–0.85)	0.70 (0.40–1.00)	381.00	0.315	0.52 (0.33–0.70)	1.00 (0.52–1.30)	120.50	0.011
BCVA_post − treatment_	0.30 (0.14–0.57)	0.52 (0.30–1.00)	294.00	0.022	0.40 (0.24–0.58)	0.75 (0.43–1.30)	125.00	0.015
CMT_baseline_ (μm)	289.00 (240.00–322.25)	367.00 (269.50–525.00)	245.50	0.003	347.50 (290.75–395.50)	512.00 (356.00–587.25)	92.00	0.001
CMT_post − treatment_ (μm)	244.50 (217.75–277.25)	269.50 (238.25–387.00)	303.00	0.044	278.00 (239.25–334.00)	340.50 (295.50–456.75)	129.00	0.020
ΔCMT (μm)	15.00 (−0.75–69.25)	72.00 (28.00–136.00)	224.00	0.001	54.50 (−0.50–88.75)	82.50 (25.00–175.00)	146.00	0.057

Data are the median (interquartile range). P-values calculated by Mann–Whitney U test. Cytokine concentrations are pre–anti-VEGF treatment levels. AH, aqueous humor; HF(–), without hyperreflective foci; HF(+), with hyperreflective foci; ΔCMT, post-treatment change in central macular thickness.

Baseline BCVA for nAMD patients without HF (median, 0.61; IQR, 0.30–0.85) was not significantly different from those with HF (median, 0.70; IQR, 0.40–1.00; *P* = 0.315, Table 2). However, for PCV patients, the baseline BCVA for those without HF, 0.52 (0.33–0.70), was better than for those with HF (*P* < 0.001). The post-treatment BCVA for nAMD and PCV patients with HF, (median, 0.52 and 0.75, respectively; IQR, 0.30–1.00 and 0.43–1.30, respectively), were worse than for those without HF (*P* = 0.022 and *P* = 0.015 respectively). The baseline CMT for nAMD and PCV patients with HF, (median, 367.00 and 512.00 μm; IQR, 269.50–525.00 and 356.00–587.25, respectively), was thicker than for those without HF (*P* = 0.003 and *P* = 0.001, respectively). Following anti-VEGF treatment, the decrease in CMT (ΔCMT) for patients with HF in the nAMD and PCV groups, 72.00 (28.00–136.00) and 82.50 μm (25.00–175.00) respectively, was greater than for those without HF (*P* = 0.001 and *P* = 0.057 respectively).

### Correlation between HF and cytokines and clinical parameters in nAMD and PCV groups

In the nAMD group, HF was correlated with the concentration of VEGF in AH (*r*_*s*_ = 0.300, *P* = 0.025, Table 3) and with the CMT_baseline_ (*r*_*s*_ = 0.476, *P* < 0.001). In the PCV group, HF was not correlated with any of the cytokine concentrations (all *P* > 0.05); however it was correlated with the BCVA_baseline_ (*r*_*s*_ = 0.407, *P* = 0.007) and the CMT_baseline_ (*r*_*s*_ = 0.531, *P* < 0.001). One month after anti-VEGF injection, HF were correlated with BCVA_post − treatment_ (nAMD: *r*_*s*_ = 0.298, *P* = 0.021; PCV: *r*_*s*_ = 0.396, *P* = 0.009), CMT_post − treatment_ (nAMD: *r*_*s*_ = 0.309, *P* = 0.016; PCV: *r*_*s*_ = 0.411, *P* = 0.006), and the ΔCMT (nAMD: *r*_*s*_ = 0.457, *P* < 0.001, PCV: *r*_*s*_ = 0.307, *P* = 0.045).

**Table 3:** Correlation between HF and AH concentrations (pg/mL) of cytokines and clinical characteristics in nAMD and PCV groups.

	**nAMD group**		**PCV group**	
	***r_*s*_***	***P***	***r_*s*_***	***P***
IL-6	0.001	0.992	−0.053	0.738
VEGF	0.300	0.025	0.091	0.575
IP-10	0.161	0.220	−0.118	0.452
MCP-1	0.057	0.666	0.009	0.953
IL-8	0.094	0.480	−0.202	0.204
IL-10	0.136	0.380	0.014	0.938
BCVA_baseline_	0.213	0.102	0.407	0.007
BCVA_post − treatment_	0.298	0.021	0.396	0.009
CMT_baseline_	0.476	< 0.001	0.531	< 0.001
CMT_post − treatment_	0.309	0.016	0.411	0.006
ΔCMT	0.457	< 0.001	0.307	0.045

P-values calculated by Spearman rho test. IL-6, interleukin 6; VEGF, vascular endothelial growth factor; IP-10, interferon-inducible protein 10; MCP-1, monocyte chemotactic protein 1; IL-8, interleukin 8; IL-10, interleukin 10; BCVA, best-corrected visual acuity; logMAR, log of the minimal angle of resolution; CMT, central macular thickness; ΔCMT, post-treatment change in central macular thickness.

### Baseline differences in cytokine levels and HF between type 1 CNV and type 2 CNV in nAMD group

Different types of CNVs in nAMD had different expression patterns in the baseline AH cytokine levels and in the number of HF (Table 4). The AH levels of VEGF and IL-10 were significantly higher in type 2 CNV than those in type 1 CNV (*P* = 0.018 and *P* = 0.019, respectively). In contrast, none of the other cytokine levels, including IL-6, IP-10, MCP-1, or IL-8 were significantly different between nAMD types 1 and 2 CNVs. Moreover, the number of HF was significantly higher in type 2 CNV than that in type 1 CNV (*P* = 0.022).

**Table 4:** Baseline differences in cytokine levels and HF between type 1 CNV and type 2 CNV in nAMD.

	**nAMD type 1 CNV (*n* = 33)**	**nAMD type 2 CNV (*n* = 30)**	***Z***	***P***
IL-6, pg/ml	3.12 (1.70–5.36)	2.64 (1.91–5.66)	480.00	0.836
VEGF, pg/ml	32.09 (21.86–47.93)	51.05 (36.35–70.18)	278.00	0.018
IP-10, pg/ml	275.60 (181.59–496.67)	380.62 (219.56–474.11)	413.00	0.259
MCP-1, pg/ml	397.91 (314.72–482.16)	427.13 (318.62–490.70)	465.50	0.685
IL-8, pg/ml	9.06 (5.51–16.02)	8.52 (6.62–14.43)	442.50	0.470
IL-10, pg/ml	0.85 (0.72–1.05)	1.05 (0.90–1.39)	162.00	0.019
HF (*n*)	1 (0–3)	2.5 (1.0–6.25)	298.00	0.022

P-values calculated by Mann–Whitney U test. IL-6, interleukin 6; VEGF, vascular endothelial growth factor; IP-10, interferon-inducible protein 10; MCP-1, monocyte chemotactic protein 1; IL-8, interleukin 8; IL-10, interleukin 10; HF, hyperreflective foci.

### nAMD patients with HF were sensitive to anti-VEGF treatment

**Figure 2:**
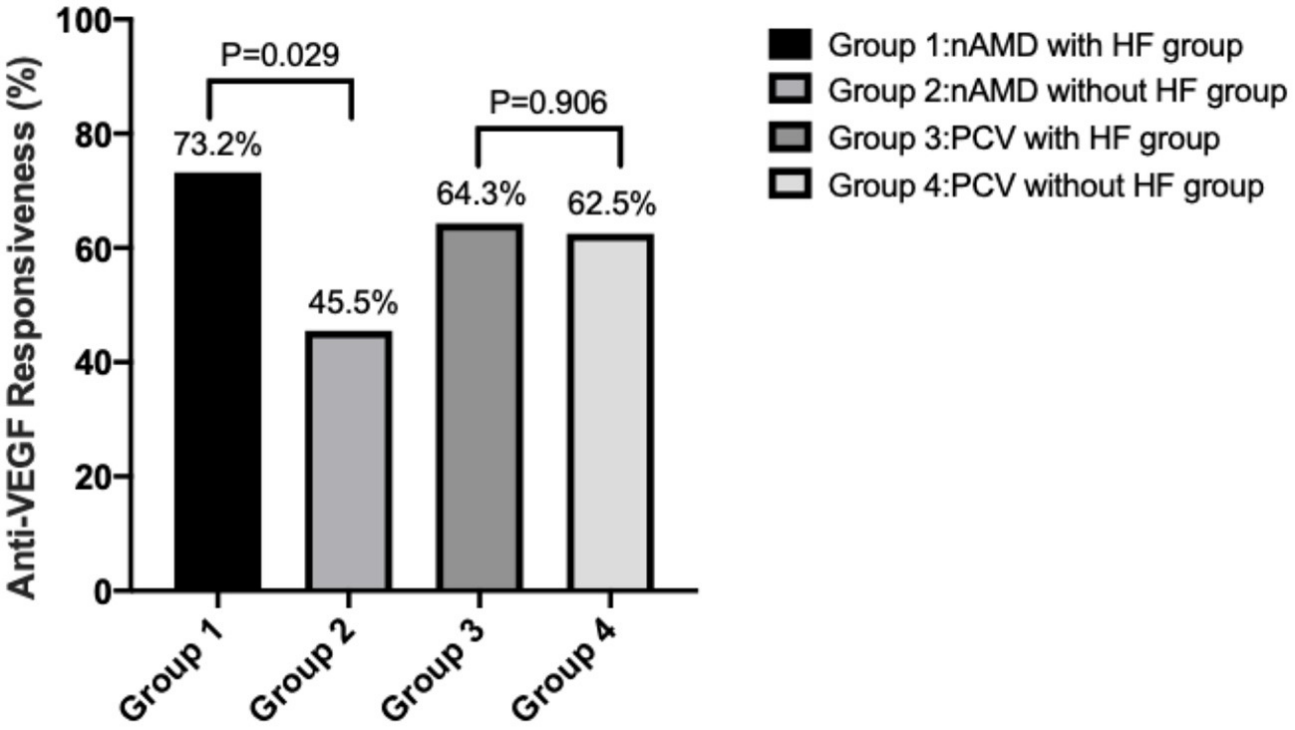
Sensitivity rate of anti-VEGF therapy in eyes with or without HF in the nAMD and PCV groups. Anti-VEGF responsiveness was calculated by CMT_reduction_ratio_.

Based on changes in the CMT_reduction_ratio_ as a measure of anti-VEGF effectiveness, eyes in which the decrease was more than 30% were considered to be sensitive to anti-VEGF treatment. At 1 month after the treatment, 30 eyes (73.2%) of the nAMD with HF group were highly responsive (Figure 2). In contrast, only 10 eyes (45.5%) of the nAMD without HF group were as responsive. Thus, nAMD eyes with HF were more responsive to anti-VEGF treatment than those without HF (*P* = 0.029). For PCV, 18 eyes with HF (64.3%) and 10 eyes without HF (62.5%) had a sensitive response to anti-VEGF treatment. Thus, there was no difference in treatment responses to anti-VEGF between PCV eyes with HF and without HF (*P* = 0.906).

## Discussion

The four most salient results emerging from this study were as follows: (1) AH levels of cytokines were differently expressed among three groups in which VEGF, IP-10, and IL-10 levels were higher in both nAMD and PCV groups compared with the control group. (2) In the nAMD group with HF, the AH levels of VEGF and IL-10 were significantly higher than those without HF. And the AH levels of VEGF were positively correlated with the number of HF. After anti-VEGF treatment, nAMD and PCV patients with HF were predisposed to worse visual outcomes and a significantly greater reduction in CMT than those without HF. (3) Compared to nAMD patients with type 1 CNV, those with Type 2 CNV had higher levels of AH VEGF and IL-10. (4) nAMD patients with HF were more sensitive to anti-VEGF treatment than those without HF.

Recent studies have shown that inflammation plays a critical role in the pathogenic progression of AMD (17, 18). Inflammation is a cascade of reactions triggered by the interaction of activated immune cells and secreted cytokines. VEGF is a cytokine of special interest because in current clinical practice intravitreal anti-VEGF injection has become the mainstay of treatment for patients with nAMD and PCV (19). However, there has been no consensus regarding the relative levels of VEGF in patients with nAMD and PCV. Some studies reported that increased VEGF levels in AH were present in both nAMD and PCV patients (20). However, others reached the opposite conclusion, indicating that there were no significant differences between nAMD, PCV, and controls (21, 22). Our data showed that AH VEGF concentrations were markedly increased in patients with nAMD and PCV compared with Controls. Joo et al. (21) and Agrawal et al. (22) reached a quite different conclusion from ours possibly due to the small sample sizes of their studies. Another plausible explanation was that compared with pancreatic diseases such as proliferative diabetic retinopathy, AMD and PCV had localized pathology and limited increase in VEGF production.

In our study, AH IP-10 concentrations were elevated in eyes with either nAMD or PCV. IP-10 was known for its vascular stabilization and anti-fibrotic activity. Previous *in vitro* studies have shown that IP-10 inhibits VEGF-mediated activation of m-calpain and interferes with newly formed blood vessels through the CXCR3 signaling pathway (23). Boulday et al. (24) reported that VEGF induced over-expression of IP-10 in endothelial cells *in vitro* and *vivo*. Considering all of this evidence, it seems that the upregulation of IP-10 was a compensatory response to excessively elevated VEGF. However, this compensatory upregulation of IP-10 was not sufficient to antagonize the angiogenic effect of VEGF in the pathogenesis of nAMD and PCV.

The third chemokine that was protruding elevated in the AH of both nAMD and PCV patients was IL-10. Previous studies have shown that IL-10 promotes angiogenesis by preventing macrophages from invading the choroid or by directly polarizing macrophages to a pro-angiogenic phenotype (25). Nakamura and colleagues found that elevated IL10 levels in the elderly activated STAT3 signaling, induced alternative macrophage activation and pathological angiogenesis (26). Thus, it was not surprising that increased levels of the inflammatory cytokine IL-10 were found in the AH of patients with nAMD and PCV.

MCP-1, as a mediator of inflammation and angiogenesis, was involved in various ocular diseases, such as branch retinal vein occlusion, proliferative diabetic retinopathy, and AMD (27–29). Histopathologic findings reported that arteriosclerotic-like hyalinization of choroidal vessels was characteristic of PCV (30). Several molecular and immunological studies showed that MCP-1 may be involved in the etiology, progression, and prognosis of atherosclerosis (31). This suggested that increased MCP-1 levels may play an essential role in the pathogenesis of PCV. Some investigators have reported that MCP-1 levels in eyes with AMD were significantly higher compared with controls (21); however, others suggested that MCP-1 levels in nAMD were not significantly different from controls (22, 32). In the present study, MCP-1 was elevated in the AH of PCV patients compared with the nAMD group, but not significantly different from the control group. It may be a potential cytokine explaining the difference between nAMD and PCV. Therefore, whether MCP-1 plays a pathogenic role in nAMD and PCV requires further investigation.

Numerous studies had shown that HF originated from activated microglial cells induced by inflammatory responses (16) and was associated with many retinal diseases, including AMD (7), diabetic macular edema (8), and central serous chorioretinopathy (9). We found the AH concentrations of VEGF and IL-10 were significantly elevated in the nAMD patients with HF compared to those without HF. Further, the AH levels of VEGF were positively correlated with the number of HF. Our results supported the hypothesis that eyes with larger amounts of HF at baseline may have a worse inflammatory reaction and more active choroidal neovascularization (33). And severe inflammatory responses are often accompanied by persistent changes in vascular permeability, so the increase in the amount of HF may reflect persistently high VEGF levels. After anti-VEGF treatment, patients with HF in either nAMD or PCV were predisposed to a worse visual outcome but had a significant reduction in CMT. A reasonable explanation for these findings is that anatomical restoration preceded functional improvement during 1 month after the anti-VEGF therapy. Segal et al. reported that the quantity of HF was associated with worst visual outcomes at baseline and at each follow-up visit (34). They hypothesized that eyes with larger amounts of HF at the initial examination may have more severe blood-retinal barrier damage, worse inflammatory reaction, and more active choroidal neovascularization leading to a poorer visual outcome (34).

With FFA and SD-OCT, the different types of nAMD CNVs can be differentiated from one another (35). Type 1 CNV is localized within the sub-RPE space, and type 2 CNV is present within the subretinal space. The VEGF and IL-10 concentrations were higher in the AMD type 2 CNV than in type 1 CNV. Because the AMD type 2 CNV existed above the RPE, cytokines secreted by them could likely spread easily within the vitreous and AH.

Based on the CMT_reduction_ratio_, nAMD patients with HF were more likely to be sensitive to anti-VEGF treatment compared to those without HF. This finding was consistent with Hsia, who found that the eyes with decreased HF after anti-VEGF treatment might have better visual acuity (36). Thus, it appears that HF might be a biomarker for predicting the effects of anti-VEGF treatment.

There are three limiting factors in our study. The first was the heterogeneity of the data (mixed treatment with ranibizumab and conbercept). Second, the manual counting of HF was time-consuming and lacks precision. Future automated software may be applied for more efficient and precise HF counting. Third, the AH samples maybe not be as valuable as vitreous fluid for detecting cytokine concentrations at the site of pathology in the retina and choroid. However, collecting vitreous samples is more invasive than collecting AH, and therefore not ethically justified for research on human subjects.

In summary, our study confirmed that SD-OCT structural parameters accompanied by differences in AH cytokine concentrations were involved in the pathogenesis of nAMD and PCV. nAMD patients with HF were more likely to respond favorably to anti-VEGF treatment than those without HF. HF could be used as an inflammatory biomarker and a predictive factor for the treatment efficacy in patients with nAMD.

## Data availability statement

Raw data supporting this study's findings will be made available from the authors upon reasonable request, without undue reservation.

## Ethics statement

All procedures involving human participants were in accordance with the ethical standards of the institutional and the National Research Committee and with the 1964 Helsinki Declaration and its later amendments or comparable ethical standards. The study was approved by the Ethics Committee of The Eye Hospital of Wenzhou Medical University. All patients provided written informed consent for inclusion in the study.

## Author contributions

JM, YijC, and LS have contributed to conceptualizing, designing the study, and final revision of the manuscript. NC and SZ have contributed to performing the experiment, data analysis, and writing. YF, ZZ, and YiqC have involved in data collection. SW and XY have organized figures and reference revisions. All authors listed contributed to the manuscript with intellectual input and approved the final version for publication.

## Funding

This work was financially supported by the Key Project of Zhejiang Medical Science and Technology Plan (WKJ-ZJ-2035) and Medical and Health Science Technology Project of Zhejiang Province (2022KY088).

## Conflict of interest

The authors declare that the research was conducted in the absence of any commercial or financial relationships that could be construed as a potential conflict of interest.

## Publisher's note

All claims expressed in this article are solely those of the authors and do not necessarily represent those of their affiliated organizations, or those of the publisher, the editors and the reviewers. Any product that may be evaluated in this article, or claim that may be made by its manufacturer, is not guaranteed or endorsed by the publisher.
