# Supplementary material for: Association between inflammatory cytokines in the aqueous humor and hyperreflective foci on optical coherence tomography in patients with neovascular age-related macular degeneration and polypoidal choroidal vasculopathy
Source: Front Med (Lausanne). 2022 Sep 23;9:973025. doi: 10.3389/fmed.2022.973025 (PMC9538653; doi:10.3389/fmed.2022.973025)
Supplement: Supplementary file 1 [file Table_1.DOCX]

Table S1. Comparisons of Cytokines levels in different gender groups.

|  | Male (n=86), Median (IQR) | Female (n=64), Median (IQR) | P |
| --- | --- | --- | --- |
| IL-6, pg/ml | 2.92 (1.72-4.95) | 4.09 (2.19-7.38) | 0.143 |
| VEGF, pg/ml | 36.23 (23.41-51.78) | 33.97 (23.57-46.07) | 0.436 |
| IP-10, pg/ml | 339.72 (217.64-520.64) | 249.99 (129.78-476.55) | 0.069 |
| MCP-1, pg/ml | 421 (346.79-505.56) | 434.80 (332.24-581.23) | 0.808 |
| IL-8, pg/ml | 9.06 (5.75-13.05) | 9.77 (6.24-15.78) | 0.505 |
| IL-10, pg/ml | 0.88 (0.63-1.05) | 0.85 (0.63-1.11) | 0.329 |

P-values calculated by Mann–Whitney U test. IL-6 = interleukin 6; VEGF = vascular endothelial growth factor; IP-10 = interferon-inducible protein 10; MCP-1 = monocyte chemotactic protein 1; IL-8 = interleukin 8; IL-10 = interleukin 10;

|  | Male | | |  | Female | | |  |  |
| --- | --- | --- | --- | --- | --- | --- | --- | --- | --- |
|  | nAMD group | PCV group | Control  group | Total | nAMD group | PCV group | Control  group | Total | P |
| IL-6, pg/ml | 2.57 (1.69-4.39) | 2.85 (1.70-5.02) | 3.09 (1.50-5.58) | 2.92 (1.72-4.95) | 4.05 (3.07-6.32) | 3.06 (2.19-4.94) | 5.22 (1.97-9.69) | 4.09 (2.19-7.38) | 0.143 |
| VEGF, pg/ml | 38.60 (22.37-61.47) | 37.68 (27.20-50.06) | 25.66 (20.44-34.18) | 36.23 (23.41-51.78) | 43.77 (28.02-63.69) | 39.23 (23.63-46.16) | 30.87 (22.01-36.26) | 33.97 (23.57-46.07) | 0.436 |
| IP-10, pg/ml | 339.12 (208.58-485.42) | 484.99 (340.51-699.87) | 143.81 (121.22-238.79) | 339.72 (217.64-520.64) | 314.39 (194.22-511.51) | 745.94 (301.31-1269.50) | 160.22 (93.74-263.03) | 249.99 (129.78-476.55) | 0.069 |
| MCP-1, pg/ml | 415.57 (309.31-495.41) | 470.57 (392.09-574.64) | 392.76 (324.80-485.09) | 421 (346.79-505.56) | 429.90 (324.73-475.53) | 572.24 (390.64-977.93) | 433.51 (332.37-585.41) | 434.80 (332.24-581.23) | 0.808 |
| IL-8, pg/ml | 8.03 (5.82-16.47) | 9.18 (7.27-11.80) | 7.93 (4.88-9.96) | 9.06 (5.75-13.05) | 9.77 (6.70-15.37) | 18.68 (7.76-39.47) | 8.65 (4.95-14.47) | 9.77 (6.24-15.78) | 0.505 |
| IL-10, pg/ml | 0.99 (0.72-1.05) | 0.88 (0.78-1.11) | 0.60 (0.47-0.69) | 0.88 (0.63-1.05) | 0.91 (0.79-1.33) | 1.33 (0.88-1.86) | 0.63 (0.57-0.88) | 0.85 (0.63-1.11) | 0.329 |
| BCVA (logMAR) |  |  |  |  |  |  |  |  |  |
| CMT (μm) |  |  |  |  |  |  |  |  |  |
| HF (n) |  |  |  |  |  |  |  |  |  |
