# Supplementary material for: Association between inflammatory cytokines in the aqueous humor and hyperreflective foci on optical coherence tomography in patients with neovascular age-related macular degeneration and polypoidal choroidal vasculopathy
Source: Front Med (Lausanne). 2022 Sep 23;9:973025. doi: 10.3389/fmed.2022.973025 (PMC9538653; doi:10.3389/fmed.2022.973025)
Supplement: Supplementary file 2 [file Table_2.DOCX]

Table S2 Differences in CMT (μm) in different drugs use in each group.

|  | **nAMD group** | |  | **PCV group** | |  |
| --- | --- | --- | --- | --- | --- | --- |
|  | **Ranibizumab**  **(n=36)** | **Conbercept**  **(n=27)** | **P** | **Ranibizumab (n=28)** | **Conbercept (n=16)** | **P** |
| CMT_baseline_ (μm) | 316.00  (259.00-395.25) | 340.00  (268.00-517.00) | 0.442 | 469.5  (347.3-561.5) | 354.0  (283.0-535.8) | 0.196 |
| CMT_post-treatment_  (μm) | 255.50  (235.75-303.50) | 261.00  (221.00-378.00) | 0.915 | 336.0  (278.5-449.3) | 309.5  (252.0-355.8) | 0.184 |
| △CMT (μm) | 37.00  (5.25-94.75) | 76.00  (28.00-119.00) | 0.147 | 75.5  (24.25-122.5) | 61.0  (3.0-193.0) | 0.938 |
| CMT_reduction_ratio_ | 0.55  (0.08-1.02) | 0.44  (-0.13-1.05) | 0.812 | 0.41  (0.13-0.83) | 0.53  (0.13-0.93) | 0.786 |

P-values calculated by Mann–Whitney U test. CMT = central macular thickness; ΔCMT = post-treatment change in central macular thickness; CMT_reduction_ratio_ = $\frac{(CMTbaseline - CMTpost-treatment)}{CMTbaseline-250\mu m}$
